# Recent progress in reactivity study and synthetic application of N-heterocyclic phosphorus hydrides

**DOI:** 10.1093/nsr/nwaa253

**Published:** 2020-09-30

**Authors:** Jingjing Zhang, Jin-Dong Yang, Jin-Pei Cheng

**Affiliations:** Center of Basic Molecular Science, Department of Chemistry, Tsinghua University, Beijing 100084, China; Center of Basic Molecular Science, Department of Chemistry, Tsinghua University, Beijing 100084, China; Center of Basic Molecular Science, Department of Chemistry, Tsinghua University, Beijing 100084, China; State Key Laboratory of Elemento-Organic Chemistry, College of Chemistry, Nankai University, Tianjin 300071, China

**Keywords:** N-heterocyclic phosphine, diazaphospholene, hydride transfer, metal-free reduction, σ-bond metathesis

## Abstract

N-heterocyclic phosphines (NHPs) have recently emerged as a new group of promising catalysts for metal-free reductions, owing to their unique hydridic reactivity. The excellent hydricity of NHPs, which rivals or even exceeds those of many metal-based hydrides, is the result of hyperconjugative interactions between the lone-pair electrons on N atoms and the adjacent σ^*^(P–H) orbital. Compared with the conventional protic reactivity of phosphines, this umpolung P–H reactivity leads to hydridic selectivity in NHP-mediated reductions. This reactivity has therefore found many applications in the catalytic reduction of polar unsaturated bonds and in the hydroboration of pyridines. This review summarizes recent progress in studies of the reactivity and synthetic applications of these phosphorus-based hydrides, with the aim of providing practical information to enable exploitation of their synthetically useful chemistry.

## Teaser

The recent progress in reactivity studies and synthetic applications of N-heterocyclic phosphorus-based hydrides was summarized to provide the basis for exploitation of their synthetically useful chemistry.

## INTRODUCTION

The hydricity of an X–H bond is defined as its propensity to transfer a hydrogen atom along with an electron. This X–H bond cleavage can enable the reduction of various unsaturated substrates such as carbonyl compounds, CO_2_, imines, olefins and aromatics [1–5]. The corresponding hydride reagents are usually categorized as metal or organic hydrides, depending on whether the hydrogen atom is bound to a metal or a nonmetal. Metal hydrides, particularly those of noble metals, have a research history of over half a century [1–3,6,7]. They are versatile reductants and have been widely used in numerous reduction processes in both academia and industry [8–11]. However, because of concerns associated with their low abundance, high toxicity and harmful transition-metal residues, considerable research has recently focused on the use of organic counterparts as surrogates for conventional metal hydrides.

**Scheme 1.**
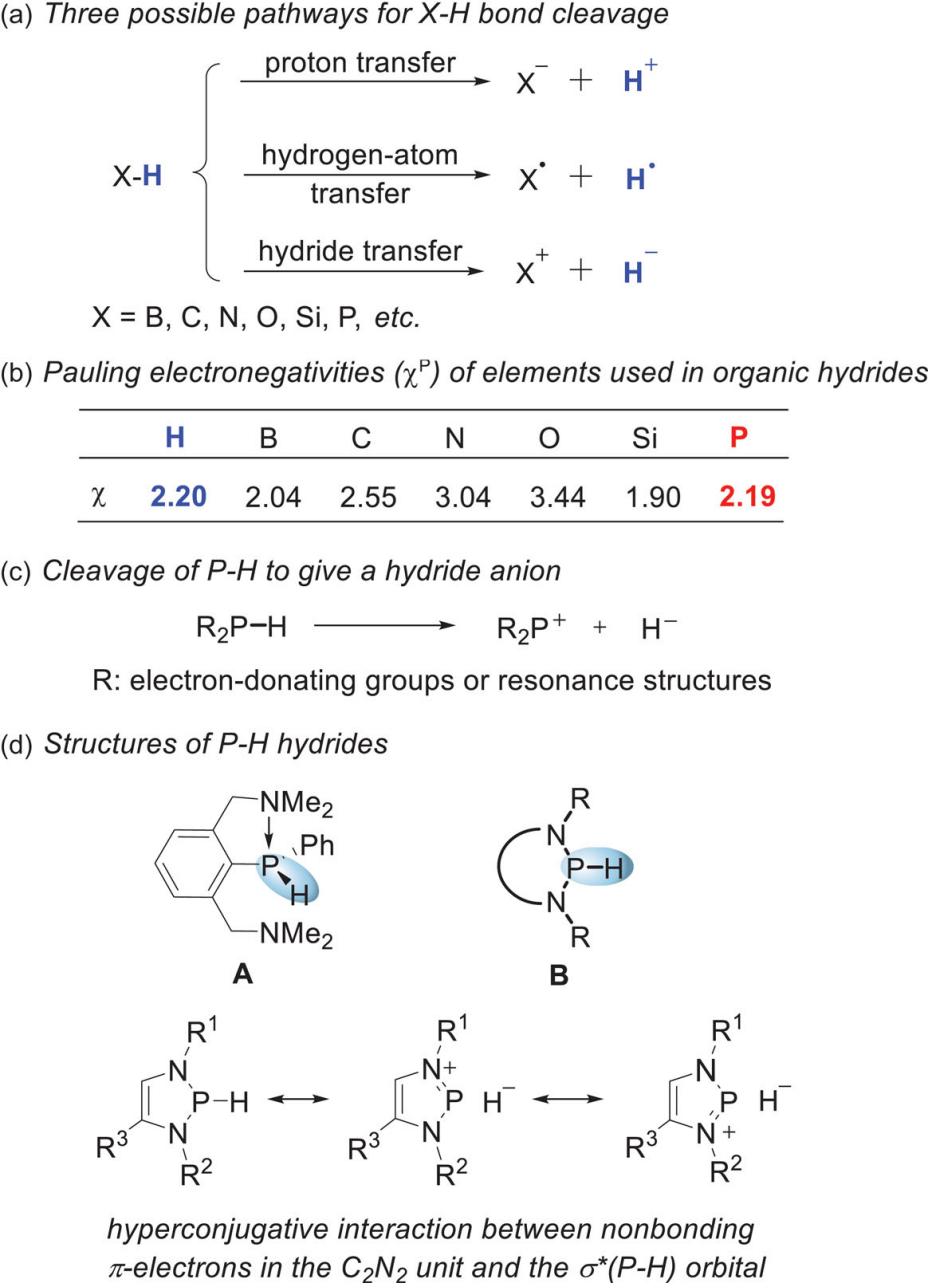
Considerations in developing P–H hydrides. (a) Three possible pathways for P–H bond cleavage. (b) Pauling electronegativities (*χ*^P^) of elements used in organic hydrides. (c) Cleavage of P–H to give a hydride ion. (d) P–H hydride structures.

Inspired by enzymatic cofactors such as nicotine adenine dinucleotide (NADH) and flavin adenine dinucleotide, various organic hydrides have been developed and used in many reduction reactions [12,13]. In this process, the design of novel structural frameworks with enhanced hydricities has attracted much attention. Generally, an X–H bond can be cleaved via three possible pathways: heterolysis to deliver a proton (H^+^), heterolysis to deliver a hydride (H^−^) and homolysis to yield a hydrogen atom (H^•^) (Scheme 1a). Choice of the reaction pathway is primarily dictated by the polarity of the particular X–H bond, i.e. the differences between the Pauling electronegativities (*χ*^P^) of the hydrogen and attached element X [12]. Scheme 1b shows the electronegativities of some typical elements. The electronegativities of boron and silicon atoms (*χ*^P^ = 2.04 and 1.90, respectively) are lower than that of the hydrogen atom (*χ*^P^ = 2.20); therefore, boron and silicon are good candidates for developing hydride reagents [12,14]. Moreover, C–H bonds can be made less protic by introducing electron-rich or -resonant motifs to polarize the C–H bond or facilitate positive-charge delocalization in conjugated hydride acceptors. These beneficial effects help to explain why NADH and triphenylmethane analogs [15,16] enable preferential release of a hydride over a proton under certain conditions. However, compared with the rapid progress made in the hydride chemistries of the elements mentioned above, exploration of P–H hydricity is lagging behind. Scheme 1b shows that the electronegativities of hydrogen and phosphorus atoms are similar. This suggests that the P–H bond is not highly polar. This paves the way for the use of P–H bonds in the construction of novel hydride donors. The described structure–hydricity relationship suggests that installing electron-donating or -resonant groups R into a P–H moiety (Scheme 1c) is an important method for improving P–H hydricity.

**Scheme 2.**
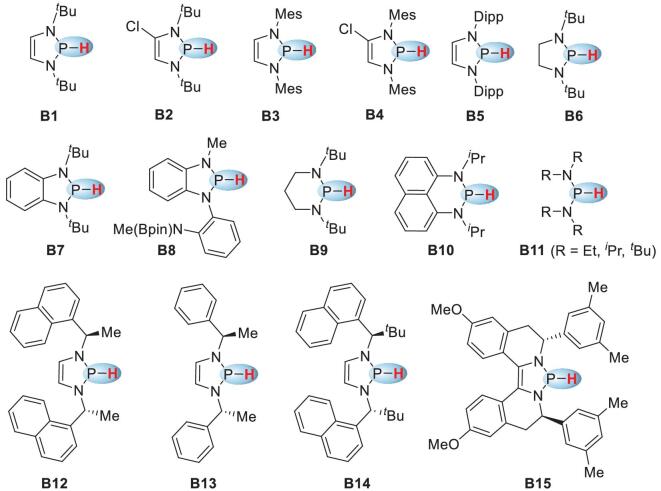
Reported phosphorus species with hydridic reactivity.

Substituent-induced P–H hydridic reactivity was first reported in 1997. It involved the reaction of a pincer-type phosphine **A** (Scheme 1d) with a trityl cation. The P–H hydricity of **A** stems from hypercoordination of the phosphorus atom with the electron-donating dimethylamino group [17,18]. Significant progress was not made until the work of Gudat and coworkers in 2000. They synthesized a series of *P*-hydrido-1,3,2-diazaphospholenes **B**, which were the first reported examples of three-coordinated phosphines featuring hydridic reactivity (Scheme 1d) [19,20]. The N-heterocyclic skeleton endows the P–H bond with excellent hydricity via a hyperconjugative interaction between the nonbonding π-electrons in the C_2_N_2_ unit and the σ^*^(P–H) orbital, as reflected by the resonance structures of the diazaphospholene skeleton in Scheme 1d. The umpolung reactivities of phosphines derived in this way stimulated researchers to identify innovative structures with comparable or enhanced hydricities. Significant developments in the past decade have made P–H hydrides an attractive research area in main-group hydrides. Many N-heterocyclic phosphines (NHPs, Scheme 2) with diverse structures and reactivities have been developed and used as powerful stoichiometric or catalytic reductants in organic syntheses.

Several exhaustive reviews have previously discussed carbon- and borane-based hydrides in terms of their hydridic reactivities and applications in chemical transformations [12,13]. However, there have been few summaries of up-to-date synthetic applications of NHPs, particularly with regard to recent progress in asymmetric chemistry [21]. Here, we outline significant advances in this area. In the next section, we describe experimental methods for quantifying the thermodynamic and kinetic hydricities of NHPs, along with a brief introduction to the NHP catalytic mechanism. The synthetic uses of NHPs as hydridic catalysts, categorized by the identity of the terminal reductants, are summarized. Where applicable, the use of measured reactivity parameters to rationalize these catalytic reductions is attempted. A brief introduction to recent progress in radical reactions of NHPs is also provided. The final section discusses promising future applications of P–H hydrides in various fields.

## QUANTIFICATION OF NHP HYDRICITY AND MECHANISTIC ANALYSIS

**Scheme 3.**
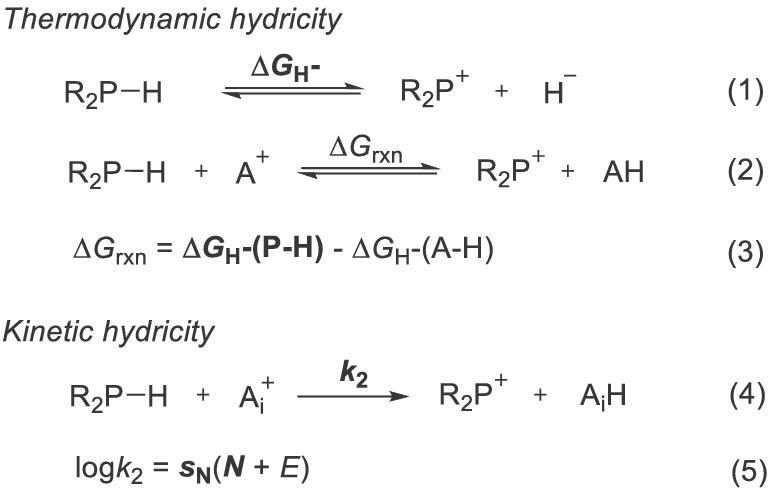
Thermodynamic and kinetic hydricities of R_2_PH.

Knowledge of the reactivity of reagents is important in designing new relevant transformations and understanding reaction mechanisms. The hydridic reactivity of NHPs was initially deduced empirically from their instability in protic and chlorinated solvents or from their fast hydride transfer to various electrophilic substrates at ambient temperature [20]. A quantitative comparison of their hydridic reactivities was then made on the basis of thermodynamic and kinetic parameters. As shown in equation (1) (Scheme 3), the thermodynamic hydricity (Δ*G*_H_−) is defined as the standard Gibbs free energy change for dissociation of a hydride donor R_2_PH to generate its conjugated hydride acceptor R_2_P^+^ and a hydride H^−^. The value of Δ*G*_H_−, i.e. the hydricity, can be obtained either by direct experimental measurements or by density functional theory (DFT) calculations. A general method for hydricity determination involves establishing an equilibrium between an unknown R_2_PH and a hydride acceptor **A^+^** of known hydricity [Δ*G*_H_−(**A**–H)] in certain media. The hydricity of R_2_PH can then be deduced from equations (2) and (3) [12,22]. However, because three-coordinated phosphines are often air and moisture sensitive, an open-flask normal isothermal titration calorimetry may not be applicable. In such case, DFT calculations provide a practical alternative. The kinetic hydricity, which is described by the empirical nucleophilicity parameter *N* and nucleophile-specific sensitivity parameter *s*_N_, is derived from the three-parameter relationship log *k*_2_ = *s*_N_(*N* + *E*) (equations (4) and (5)), also known as the Mayr equation. It can be used as an alternative to the thermodynamic hydricity [23–25]. Such nucleophilicity parameters are obtained from the second-order rate constants *k*_2_ of hydride transfer between a nucleophile and reference acceptors **A***_i_* (*i* = 1, 2, 3, …) with known electrophilicity parameters *E*. These parameters can be used to quantify the hydridic reactivity of an NHP and to guide rational selection of suitable NHPs for diverse substrates in reaction design.

### Kinetic hydricities of NHPs

In our recent work, we used the Mayr equation to establish an NHP kinetic hydricity scale (Table 1) [26]. The NHPs examined covered those frequently used in organic syntheses; **B9** was included for comparison. The data in Table 1 show that the nucleophilicity parameters *N* of NHPs in MeCN span a broad range, namely 13.5–25.5. Among these, **B1** (*N* = 25.5) is the most nucleophilic hydride donor ever quantified by the Mayr equation. It has a nucleophilicity far stronger than that of NaBH_4_ (*N* = 14.74 in DMSO). Therefore, **B1** can be regarded as a superhydride donor, which can reduce inert CO_2_ without activators (see the applications’ discussion). When the tertiary butyl groups in **B1** are replaced with electronically different, bulkier aryl groups, such as in the 2,4,6-trimethylphenyl analog **B3** and 2,6-diisopropylphenyl analog **B5**, the corresponding *N* values (*N* = 17.68 and 19.85, respectively) decrease to a level close to that of **B6** (*N* = 18.74). This clearly shows that the kinetic hydricity is sensitive to variations in steric hindrance around the P–H bond. The benzannulated moiety in **B7** attenuates the hydricity via delocalization of the lone-pair electrons on N atoms toward the phenyl ring. As expected, **B9** and **B10** [27] show the weakest hydricities (*N* = 13.46 and 8.64, respectively) among the P–H reagents examined. However, they still have nucleophilicities stronger than, or similar to, those of many other commonly used organic hydrides [28] such as arylbenzimidazolines (*N* = 9.72–10.14 in MeCN) [29] and dihydropyridines (*N* = 7.53–9.00 in CH_2_Cl_2_) [30]. The nucleophile-specific sensitivity parameters (*s*_N_) are small (Table 1, *s*_N_ = 0.35–0.68) and similar to those observed for other extremely reactive nucleophilic systems (e.g. N-heterocyclic carbenes). This reflects their low sensitivity to changes in electrophiles [31–34]. In combination with the electrophilic parameters of the hydride acceptors, these nucleophilicity parameters can be used to evaluate the feasibility of hydride transfer and to differentiate NHP reactivities toward different substrates. This will be further addressed in the synthesis part, if the necessary data are available.

**Table 1.** Nucleophilicity parameters for NHPs in MeCN and other commonly used hydride reagents for comparison (Mes = 2,4,6-trimethylphenyl and Dipp = 2,6-diisopropylphenyl).

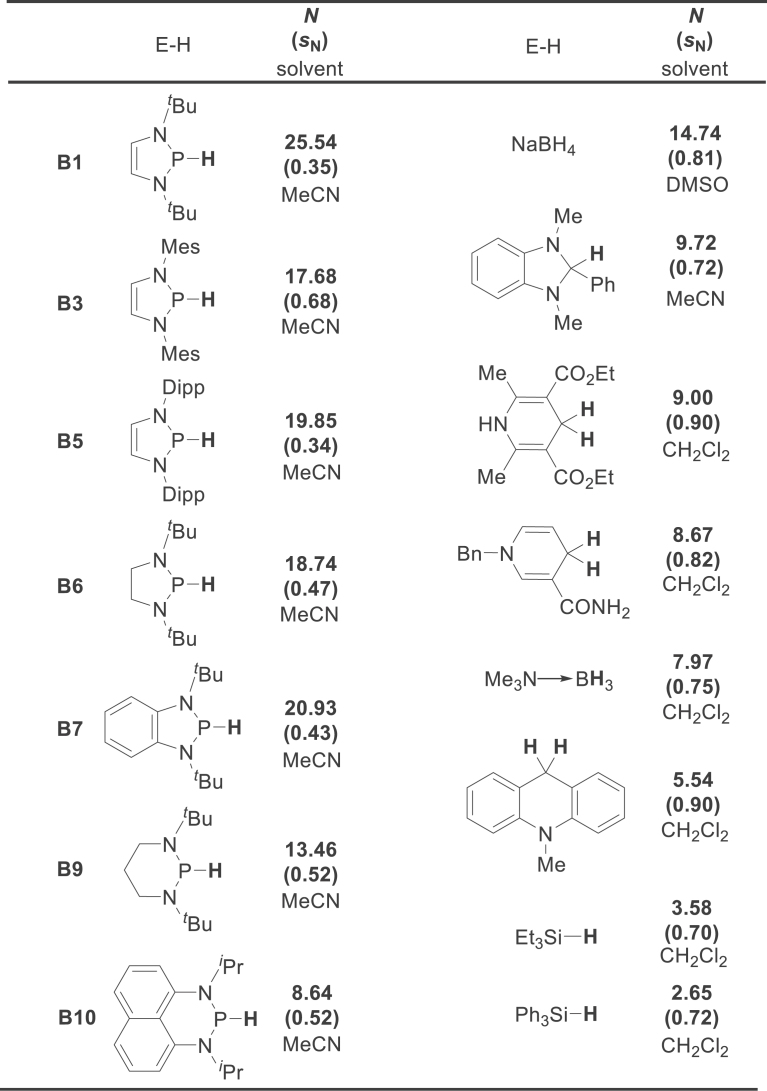		

### Thermodynamic hydricities of NHPs

Our group used an equilibrium method to determine the thermodynamic hydricities (Δ*G*_H_−) of **B9** and **B10** in MeCN experimentally for the first time; the values are 48.8 and 62.2 kcal mol^−1^, respectively [27]. This is in line with the kinetic results. These results again show that even the weakest nucleophile, i.e. **B9**, in Table 1 has a hydricity comparable to those of the conventional strong hydride donors NaBH_4_ (Δ*G*_H_− = 50 kcal mol^−1^ in MeCN) and 2,3-dihydrobenzo[*d*]imidazoles (Δ*G*_H_− = 45 kcal mol^−1^ in MeCN) [35]. The thermodynamic and kinetic investigations both showed that the P–H bond in **B10** can dissociate via all the homolytic and heterolytic pathways in Scheme 1a under appropriate conditions; therefore, it can serve as hydride, hydrogen-atom, and proton donors. The extremely negative oxidation potentials of the phosphinyl radicals of **B9** and **B10** (−2.39 and −1.94 V, respectively, versus Fc^+/0^ in MeCN) indicate their potent electron donicities. Attempts to acquire Δ*G*_H_− values for other NHPs in Table 1 have as yet been unsuccessful because of lack of suitable counterparts that enable equilibria (equation (2)) to be established. A relative hydricity (versus BEt_3_) sequence for several NHP analogs was reported by Zhao and coworkers from DFT calculations [36].

### Mechanism of NHP catalysis

**Figure 1.**
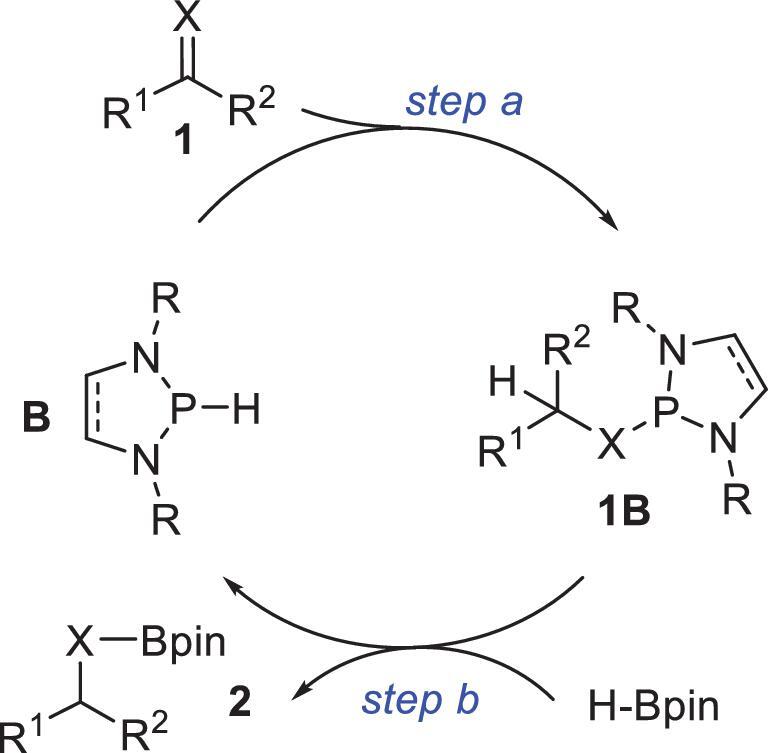
Proposed mechanism of NHP-catalyzed reduction of C–X bonds (X = O or NR) with pinacolborane (HBpin).

In principle, the mechanism of an NHP-catalyzed reduction, even a simplified one, involves at least two basic steps (Fig. 1), i.e. initial cleavage of the P–H bond to furnish intermediate **1B (**bearing a newly formed P–X bond, X = OR or NR, step a), and subsequent regeneration of the P–H bond to deliver product **2** (step b). Previous reactivity investigations have shown that NHPs have powerful hydride donicities, and this ensures smooth hydride release (step a) [20]. However, NHP regeneration (step b) may be problematic because regeneration of a highly reactive P–H bond from a P–X bond is often thermodynamically unfavorable. For this step to proceed, a concomitant process that compensates for the unfavorable thermodynamics is necessary. Considering the robustness of B–X and Si–X bonds, boranes and silanes are promising candidates for achieving the desired reactivity. This is because formation of a B–X or Si–X bond is highly exothermic, which can provide the required supplementary driving force for concurrent P–H bond formation.

**Figure 2.**
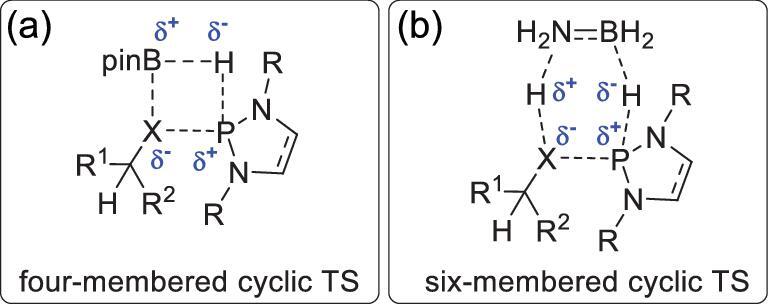
Four- (a) and six-membered (b) cyclic TSs for NHP regeneration.

In NHP-catalyzed reductions, HBpin, H_3_N–BH_3_ and sometimes Ph_2_SiH_2_ are preferentially used as terminal reductants. Several mechanistic studies of P–H bond regeneration have indicated that a concerted mechanism (σ-bond metathesis) [37] with a six- [38] or four-membered [39] cyclic transition state (TS; Fig. 2) should be considered. We categorized these reactions into two primary types: non-asymmetric and asymmetric reductions. These are discussed in the following sections.

## NON-ASYMMETRIC REDUCTIONS

### Ammonia–borane (H_3_N–BH_3_) as a reductant

**Scheme 4.**
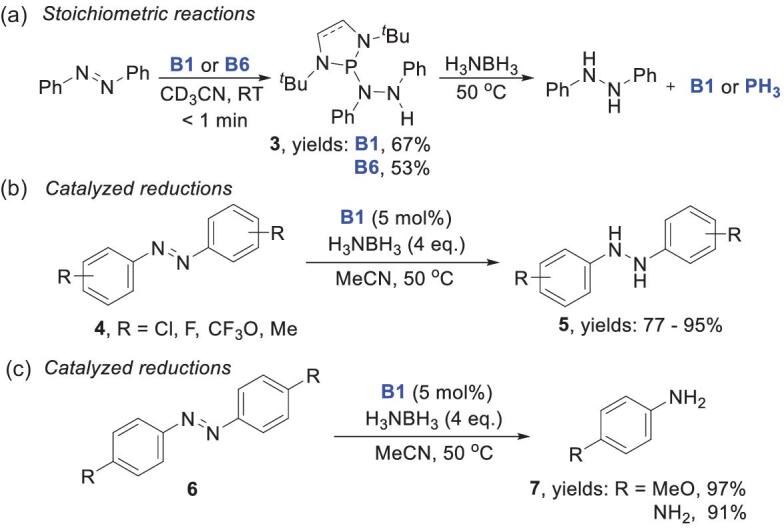
NHP-catalyzed hydrogenation of N=N bonds with H_3_N–BH_3_. (a) Stoichiometric reactions. (b, c) Catalyzed reductions.

In 2014, Kinjo and coworkers reported the first synthetic application of NHPs in catalytic hydrogenations [38]. They found that both **B1** and **B6** can reduce azobenzene smoothly at room temperature (Scheme 4a). Subsequent P–H regeneration with H_3_N–BH_3_ gave quantitative recovery of **B1**, whereas **B6** was further reduced to PH_3_. **B1** was therefore selected as the catalyst. Hydrogenation of the N=N bond in **4** with H_3_N–BH_3_ was achieved in good to excellent yields (77%–95%) (Scheme 4b). Notably, for substrates **6** with 4-NH_2_ or 4-MeO groups, subsequent cleavage of N–N bonds occurred, which gave further reduced anilines **7** (Scheme 4c). This is presumably because electron-donating substituents at the *para* position increase the reactivity of the N–N bonds in the hydrazine intermediates, which favors further reduction. Kinetic and computational studies supported a concerted double-hydrogen-transfer mechanism with a six-membered TS for the regeneration of catalyst **B1** (Fig. 2). Notably, the calculated profiles show that reduction was initiated by nucleophilic attack of the **B1** phosphorus atom on the N=N bond, rather than direct hydride transfer.

**Scheme 5.**
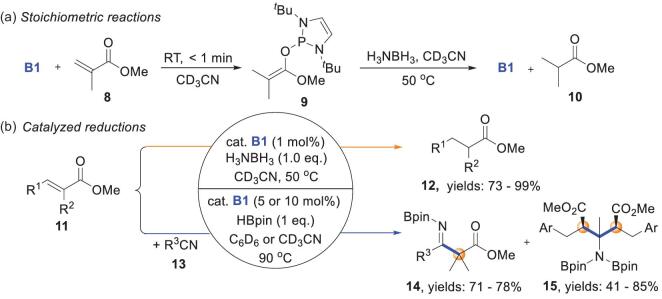
NHP-catalyzed reduction of α,β-unsaturated esters and C–C couplings with nitriles. (a) Stoichiometric reactions. (b) Catalyzed reductions.

Kinjo and coworkers later used a similar strategy for the catalytic reduction of α,β-unsaturated esters on the basis of their preliminary results for stoichiometric reactions (Scheme 5a) [40]. A combination of 1 equiv. of H_3_N–BH_3_ and 1 mol% **B1** efficiently converted α,β-unsaturated esters **11** to saturated esters **12** (Scheme 5b). It is worth noting that the nucleophilicity *N* ≈ 8 for H_3_N–BH_3_ [15] and electrophilicity *E* ≈ −20 for unsaturated esters [41] suggest that the uncatalyzed reactions should not take place. Replacing H_3_N–BH_3_ with HBpin in the presence of nitriles at an elevated temperature (90°C) led to further C–C bond coupling between boryl enolate intermediates and nitrile **13**. This afforded either single- (imine products **14**, 71%–78% yields) or double-addition (**15**, 41%–85% yields) diester derivatives, depending on whether the steric congestion of the formed boryl enolate intermediates prevented the second addition to retain the imine moiety. The similar stoichiometric and catalytic conjugate reductions have been previously reported by Gudat and coworkers [19,20] and Speed and coworkers [42], respectively.

### HBpin as a reductant

**Scheme 6.**
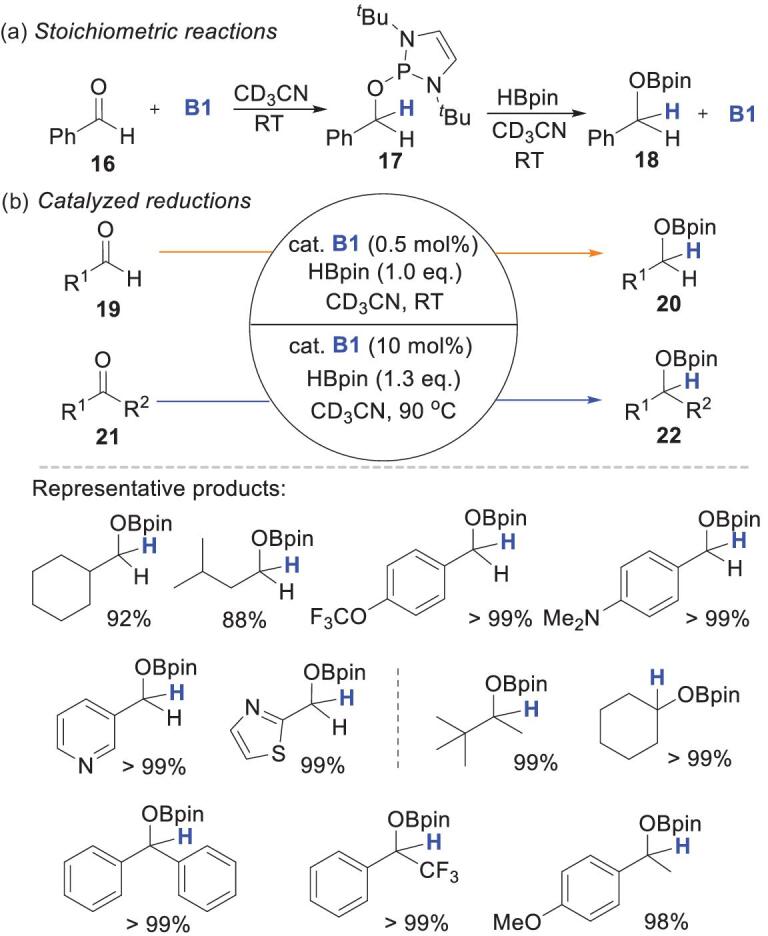
NHP-catalyzed hydroboration of carbonyl compounds. (a) Stoichiometric reactions. (b) Catalyzed reductions.

Among various hydrogen sources for NHP-catalyzed reductions, HBpin is the most common reagent because of its outstanding reaction performance, good solubility and low price. In 2015, Kinjo and coworkers reported the first metal-free catalytic hydroboration of carbonyl compounds by using 0.5–1 mol% **B1** and a stoichiometric amount of HBpin [39]. They synthesized intermediate **17** via the reaction of benzaldehyde **16** with **B1** (Scheme 6a) [20]. ^11^B and ^31^P NMR spectroscopic investigations indicated that treatment of **17** with 1 equiv. of HBpin led to the formation of **B1** and the hydroboration product **18**. This confirms the feasibility of P–H regeneration from HBpin. DFT calculations supported a four-membered cyclic TS for P–H bond formation. High catalytic activity, at a catalyst loading as low as 0.5 mol%, was observed in the reaction with aldehydes **19** (Scheme 6b). Formyl groups can be selectively reduced in the presence of heterocycles and ketones, in contrast to previously reported results, i.e. dearomatization [43] or complete reduction [44,45]. However, higher catalyst and reductant loadings (10 mol% and 1.3 equiv.) and temperature (90°C), and longer reaction times were required to achieve reduction of ketones **21** (Scheme 6b) because of their lower electrophilicity [46] and larger steric hindrance. Under the optimized conditions, both acyclic and cyclic ketones were well tolerated and furnished the corresponding borate esters quantitatively.

**Scheme 7.**
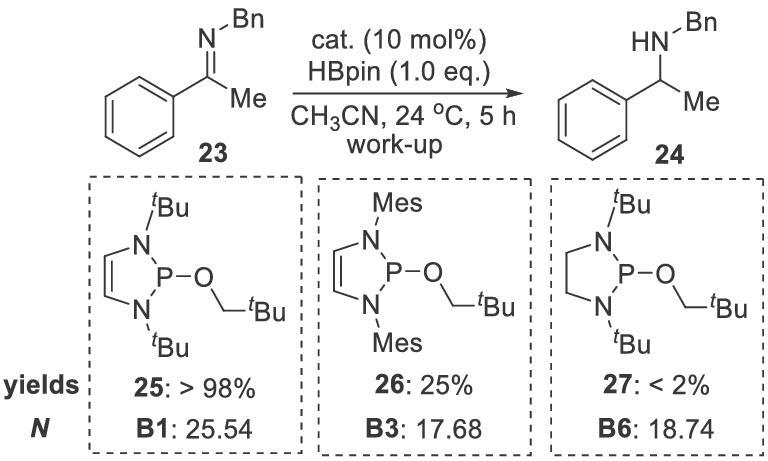
Condition optimization for alkoxydiazaphospholene-catalyzed imine reductions.

**Scheme 8.**
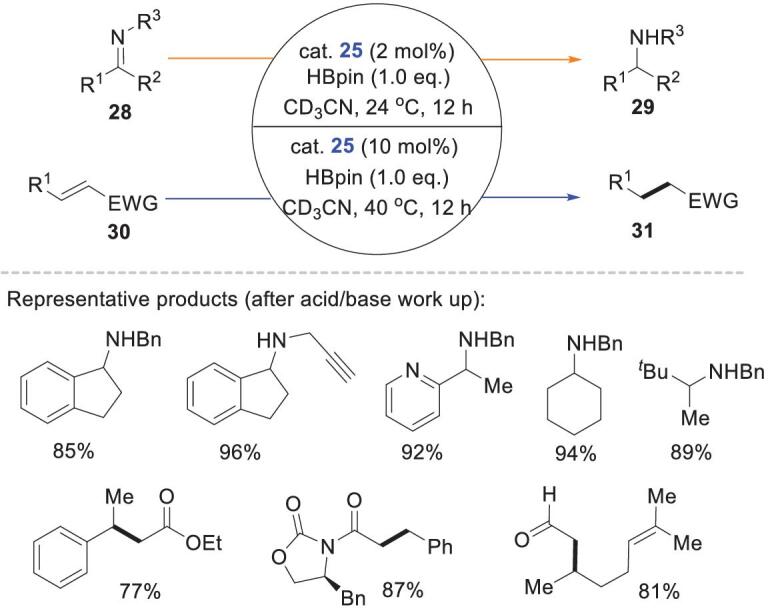
Alkoxydiazaphospholene-catalyzed imine and conjugate reductions.

To increase the moisture/oxygen tolerance of NHPs, Speed and coworkers developed alkoxydiazaphospholenes for catalytic reductions. The precatalysts **25**, **26** and **27**, which correspond to catalysts **B1**, **B3** and **B6**, respectively, are sufficiently stable for open-air operation [42]. In the catalytic reduction of imines **23** with HBpin, they gave 98%, 25% and <2% yields, respectively (Scheme 7). Precatalyst **25** gave the best performance. The worst performance, i.e. by **27**, partly resulted from failure to generate **B6** from **27** in the presence of excess HBpin. Precatalyst **25** was then used to perform imine reductions with HBpin (Scheme 8). The reaction showed good functional group compatibility, and various imines were reduced to amines in good yields. However, imines with sulfenyl or trifluoromethyl groups were not viable substrates. Perhaps these electron-withdrawing groups inhibit NHP regeneration from P–N intermediates. This system is also applicable to conjugated substrates; the corresponding products are obtained in moderate yields (Scheme 8).

**Scheme 9.**
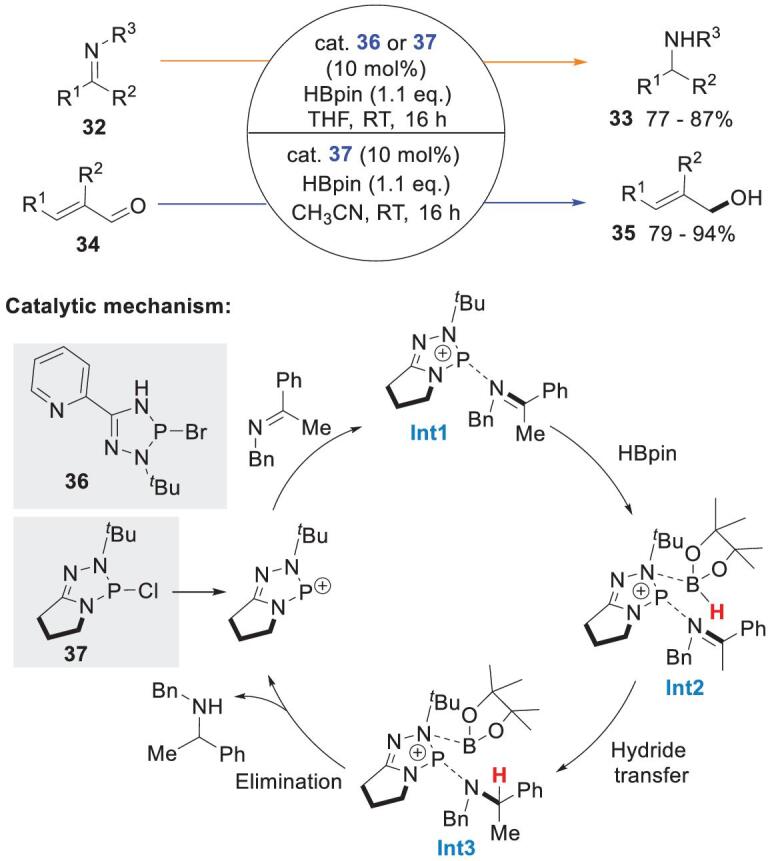
Triazaphospholene-catalyzed hydroborations of imines and α,β-unsaturated aldehydes.

The same group synthesized a series of triazaphospholenes **36** and **37**, and investigated their reactivities in reductions of imines **32** and α,β-unsaturated aldehydes **34** (Scheme 9) [47]. ^31^P NMR spectroscopy showed that P–H bonds were not formed from a mixture of **37** and HBpin in CD_3_CN. This precludes a P–H hydride transfer mechanism. Presumably, catalyst **37** was ionized to the phosphenium cation, which can combine with the imine N atom to form a van der Waals prereaction intermediate **Int1**, which then interacted with HBpin to give the intermediate **Int2**. DFT calculations showed that **Int2** was converted to the intermediate **Int3** via a six-membered TS with an activation barrier of 23.0 kcal mol^−1^. This cyclic TS may account for the high 1,2-chemoselectivity observed in unsaturated aldehyde reductions. Notably, the triazaphosphenium cations act as Lewis acids and activate the substrate imines rather than as precursors of P–H hydrides.

**Scheme 10.**
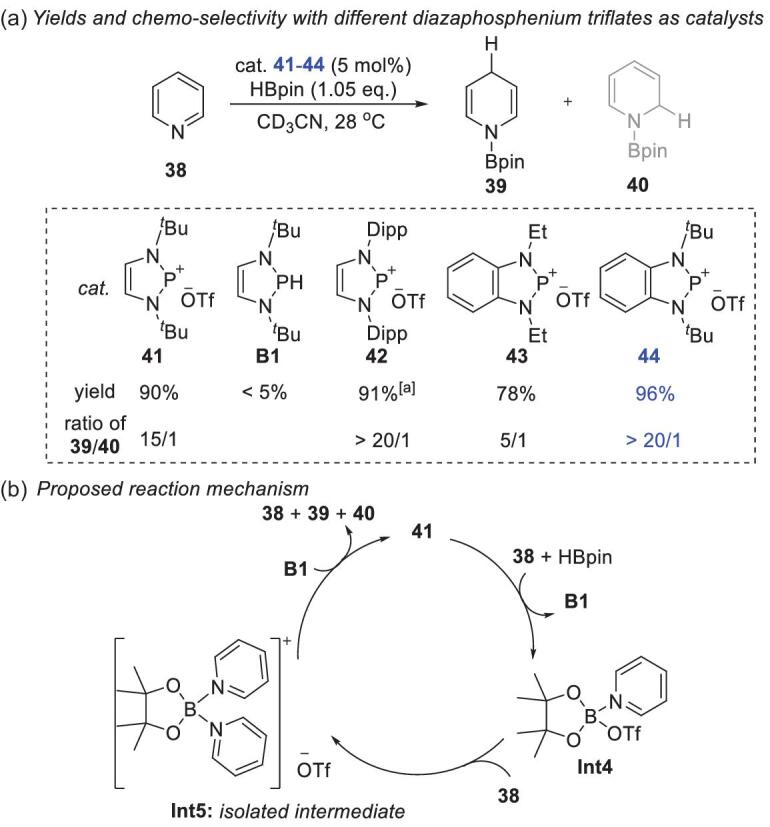
Pyridine hydroboration catalyzed by diazaphosphenium triflates. (a) Yields and chemoselectivities with different diazaphosphenium triflates as catalysts. (a) Reaction temperature: 70°C. (b) Proposed reaction mechanism.

All the reductions mentioned above focused on polar double bonds, e.g. polar olefin, imine and carbonyl substrates*.* Reduction of aromatic compounds under metal-free conditions is challenging because dearomatization is thermodynamically unfavorable. In 2018, Kinjo and coworkers reported the hydroboration of pyridines catalyzed by 1,3,2-diazaphosphenium triflates [48]. Condition optimization indicated that diazaphosphenium **44** gave the best performance (Scheme 10a). A variety of pyridines can be reduced with 5 mol% **44** to 1,4-hydroboration products, some of which are otherwise unavailable. This reaction has excellent reactivity, good selectivity and a broad substrate scope, except for pyridines with 2-Cl, 2-CN, 2,6-Me_2_ and 4-Me_2_N groups. Mechanistic studies ruled out the possibility of pyridine activation by phosphenium cations. A borane–pyridine complex was therefore considered as the active intermediate. The proposed mechanism is shown in Scheme 10b. First, **Int4** and **B1** were formed via activation of HBpin by pyridine **38** and a triflate anion. Another pyridine coordinated with **Int4** to produce **Int5**, which was isolated and spectroscopically characterized. One of the activated pyridines in **Int5** then abstracted a hydride from **B1** to complete the reduction. Speed and coworkers later reported a similar strategy for pyridine hydroboration with catalyst **25**. Unlike the broad substrate scope with **44**, Speed's system only worked for electron-deficient pyridines [49].

**Scheme 11.**
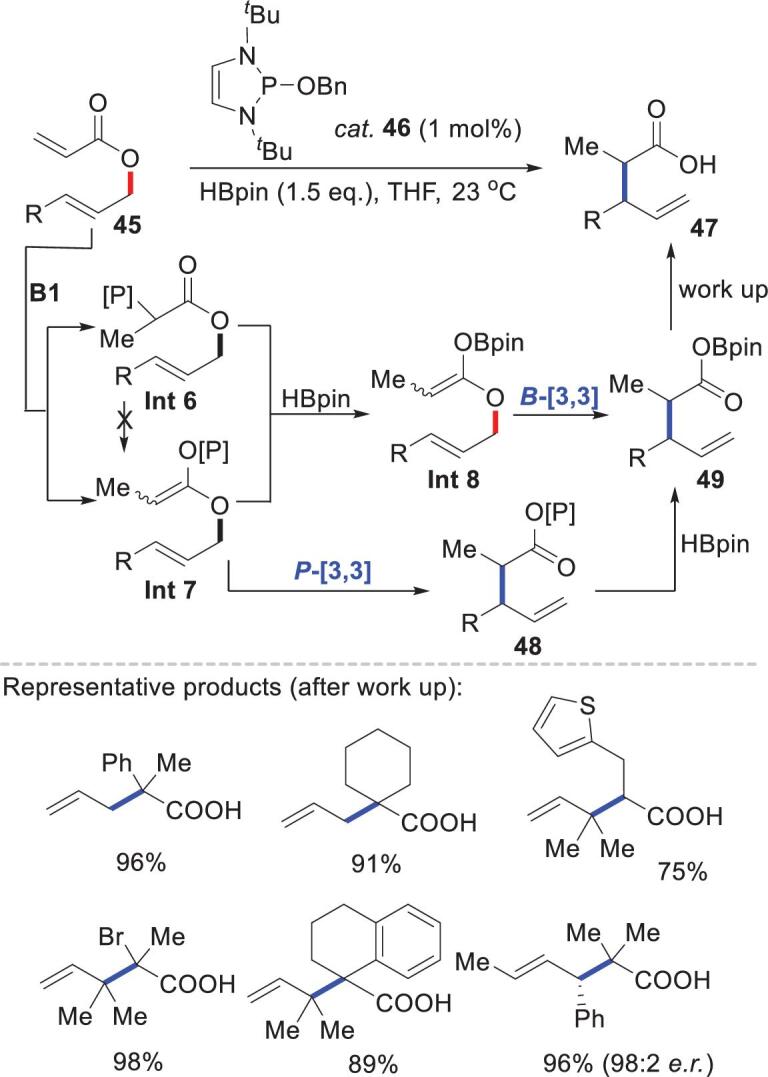
NHP-catalyzed reductive Claisen rearrangement. **B1** = [P]–H.

Hydride transfer from NHPs can also initiate Claisen rearrangement [50,51]. Cramer and coworkers developed an NHP-catalyzed reductive Claisen rearrangement for effectively constructing C–C bonds, particularly those bearing quaternary carbon centers. The reaction, which was performed at ambient temperature with 1 mol% precatalyst **46**, was compatible with a variety of functional groups (Scheme 11) [52]. Mechanistic studies suggested two possible pathways: *B*-[3,3] and *P*-[3,3] rearrangements. The initial hydride transfer between **45** and **B1** (generated *in situ* from **46** and HBpin) gave either a P–C or P–O intermediate (**Int6** or **Int7**), depending on the substrate. Both intermediates can be converted to intermediate **Int8** via a σ-bond metathesis with HBpin. The *B*-[3,3] Claisen rearrangement then directly converted **Int8** to **49**. The *P*-[3,3] pathway yielded the same product **49** via a two-step process: the Claisen rearrangement of **Int7** to **48**, and subsequent metathesis with HBpin.

**Scheme 12.**
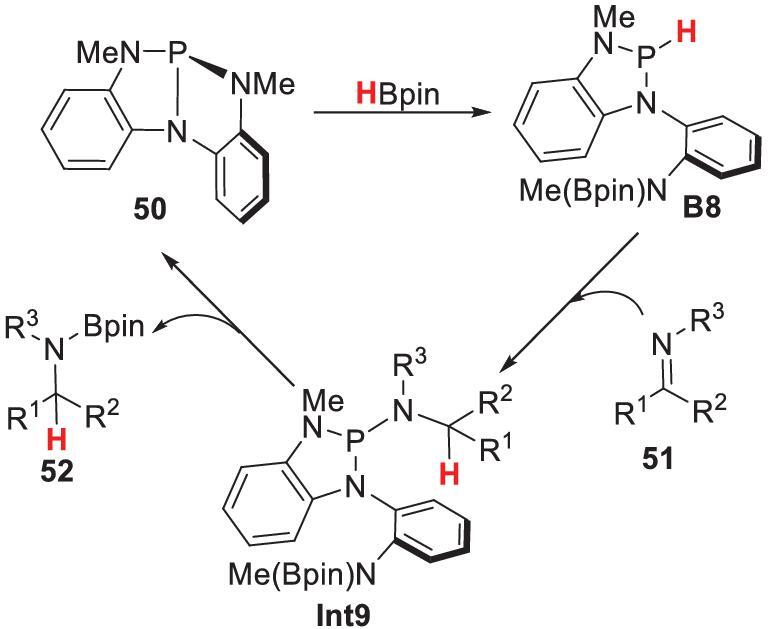
Catalytic hydroboration of imines by distorted phosphorus triamide **50**.

Examples of productive endocyclic P–N bond cleavage are rare. Recently, Radosevich and coworkers reported a geometrically deformed tricoordinated phosphorus triamide **50** [53]. This triamide reacted with HBpin to give **B8** via scission of an endocyclic P–N bond (Scheme 12). This reactivity stems from cooperation between an electrophilic phosphorus center and a proximal basic *N*-methylanilide nitrogen, i.e. a behavior similar to that of a frustrated Lewis pair. **B8** showed good hydridic reactivity and reduced imine **51** to intermediate **Int9**. Rapid intramolecular boryl transfer eliminated the *N*-borylamine **52** and catalyst **50**. This P–N ligand cooperation provides a platform for designing new constrained main-group catalysts.

**Scheme 13.**
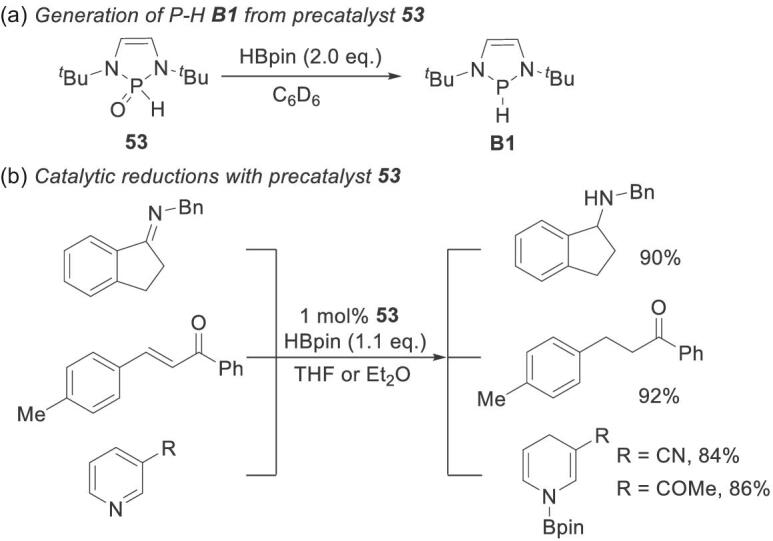
Catalytic reductions with precatalyst **53**. (a) Generation of P–H **B1** from precatalyst **53**. (b) Catalytic reductions with precatalyst **53**.

Recently, Speed and coworkers discovered that the air-stable secondary phosphine oxide (SPO) **53** was transformed into **B1** in the presence of HBpin (Scheme 13a) [54]. This bench-purifiable SPO could replace highly air-sensitive NHPs as reduction catalysts. This possibility was confirmed by performing SPO-catalyzed imine and conjugate reductions, and pyridine hydroboration, with HBpin (Scheme 13b). These results will help to overcome the limitations associated with reagent sensitivity to help popularize NHP chemistry.

### Ph_2_SiH_2_ as a reductant

**Scheme 14.**
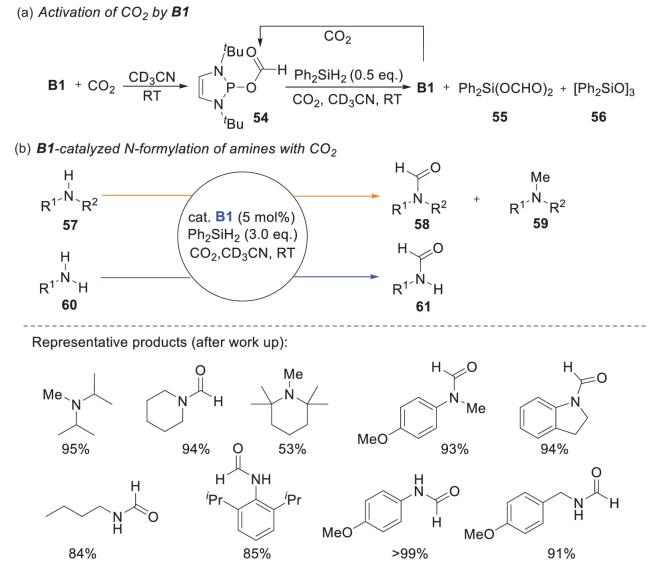
NHP-catalyzed N-formylation and N-methylation of amines with activated CO_2_. (a) Activation of CO_2_ by **B1**. (b) **B1**-catalyzed N-formylation of amines with CO_2_.

Another hydridic reductant, i.e. Ph_2_SiH_2_, can also regenerate NHPs through a σ-metathesis mechanism. An example was reported by Kinjo's group [55]. Building on the success of catalytic reduction of carbonyl groups by **B1**, the authors investigated NHP-catalyzed CO_2_ reduction. They found that exposure of **B1** to 1 atm of CO_2_ gave a hydrophosphination product, namely the phosphorus(III) formate **54**. Regeneration of catalyst **B1** was achieved by adding 0.5 equiv. of Ph_2_SiH_2_ to the reaction mixture. This furnished **B1** and electrophilic Ph_2_Si(OCHO)_2_**55**, along with siloxane **56** as a minor product (Scheme 14a). Primary and secondary amines were then examined as nucleophiles for N-formylation at a 5 mol% catalyst loading. The reaction had a broad substrate scope, which included alkyl and aryl amines (Scheme 14b). Note that sterically hindered and highly basic amines such as diisopropylamine and 2,2,4,4-tetramethylpiperidine afforded N-methylated products because of over-reduction of the corresponding *N*-formylamines [56,57]. DFT calculations performed by Ye and coworkers showed competition between formylation and methylation of amines [58].

### Mg powder as an electron donor

**Scheme 15.**
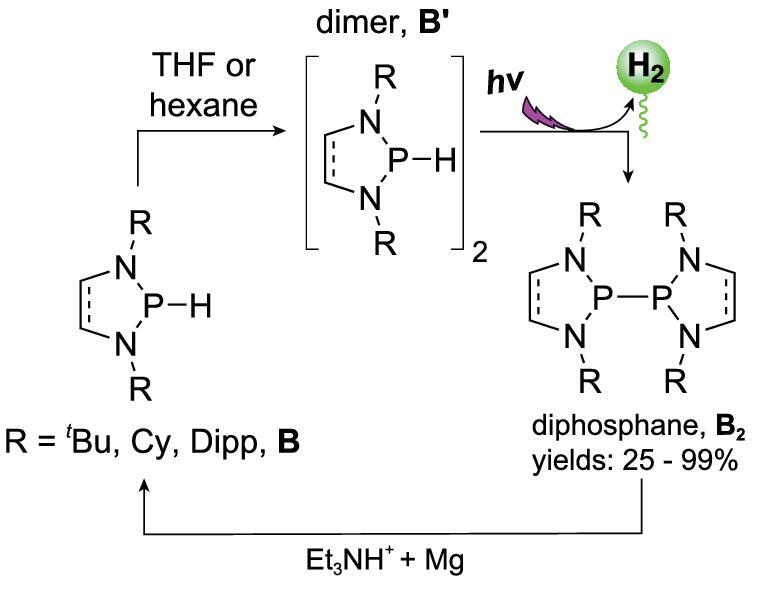
NHP-catalyzed photochemical H_2_ evolution.

In addition to single-component reductants, a combination of Mg powder as an electron donor and a Brønsted acid can also serve as a formal hydrogen donor in NHP recovery from diphosphines. Gudat and coworkers found that during NHP syntheses some of the formed NHPs were converted to diphosphine **B_2_** under ultraviolet irradiation (Scheme 15) [59]. Unlike P_2_H_4_ [60,61], these diphosphines are photostable, and can therefore be recycled in photocatalytic reactions. Based on this, Gudat explored the photocatalytic generation of H_2_ from catalyst **B** with Et_3_NH^+^Cl^−^ and Mg as the formal hydrogen source. DFT calculations showed that prior to H_2_ evolution, the key dimer **B**^**′**^ was formed. Its subsequent photochemical excitation yielded H_2_ and the diphosphine **B_2_** (Scheme 15). **B_2_** was then reduced to NHPs by the reductant pair Et_3_NH^+^Cl^−^ and Mg. This process can be regarded as an NHP-catalyzed photochemical reduction of a proton to H_2_ by Mg.

## ASYMMETRIC REDUCTIONS

The unusual reactivities of NHPs in non-asymmetric reductions have stimulated interest in the development of asymmetric variants by using chiral diamine motifs, which are versatile building blocks in the synthesis of chiral N-heterocyclic carbenes [62], diaminophosphine oxides [63] and phosphorodiamidite ligands [64]. Representative structures are shown in Scheme 2 (**B12**–**B15**). All of these retain the diazaphospholene skeleton, which is crucial for achieving excellent hydricity.

**Scheme 16.**
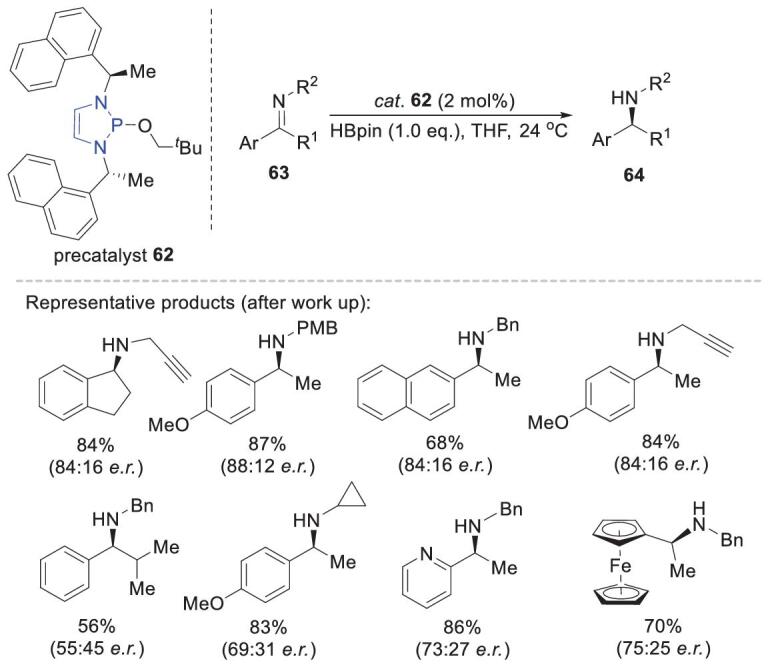
Asymmetric imine hydroboration catalyzed by chiral diazaphospholene **62**.

In 2017, Speed and coworkers reported the first use of NHPs as chiral catalysts for asymmetric reductions (Scheme 16) [65]. They reported that use of 2 mol% **62** (the precatalyst of **B12**) enabled imine reduction by HBpin, with moderate enantioselectivities (er 55 : 45 to 88 : 12). This was the best reported enantioselectivity for alkylimine hydroboration with HBpin at that time. Changing the catalyst from **B12** to **B13** or **B14** decreased the enantioselectivity. Control experiments suggested a similar mechanism to that for the non-asymmetric version.

**Scheme 17.**
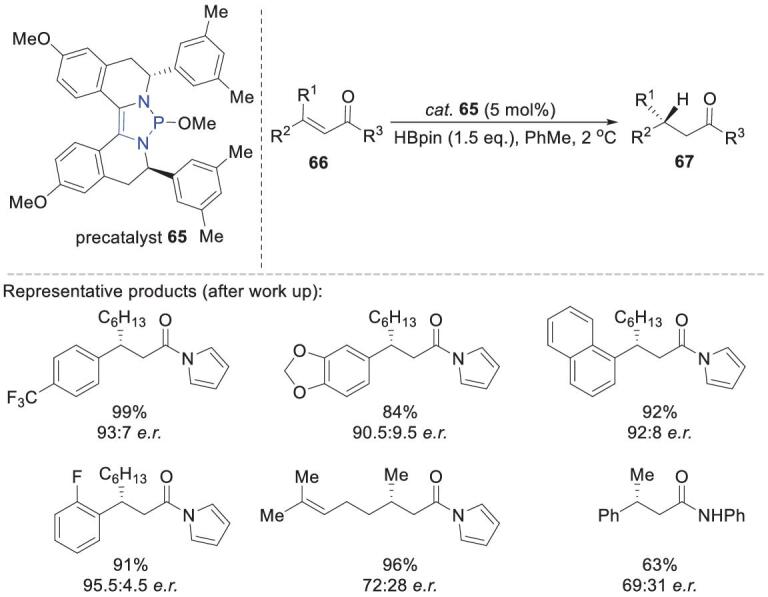
Asymmetric conjugate hydroboration catalyzed by chiral diazaphospholene **65**.

Cramer and coworkers developed a series of new chiral NHPs for performing asymmetric conjugate reductions with HBpin (Scheme 17) [66]. Initially, they used **B12** and **B13** (generated *in situ* from their corresponding precatalysts) as catalysts, but obtained moderate er values. To improve the enantioselectivity, they synthesized **65**, a precatalyst of **B15**, which has more rigid moieties around the P–H bond. Condition optimization showed that 5 mol% **65** in toluene at 2°C gave the best result. Based on this, various conjugated substrates were reduced with high enantioselectivity (er values up to 95.5:4.5).

**Scheme 18.**
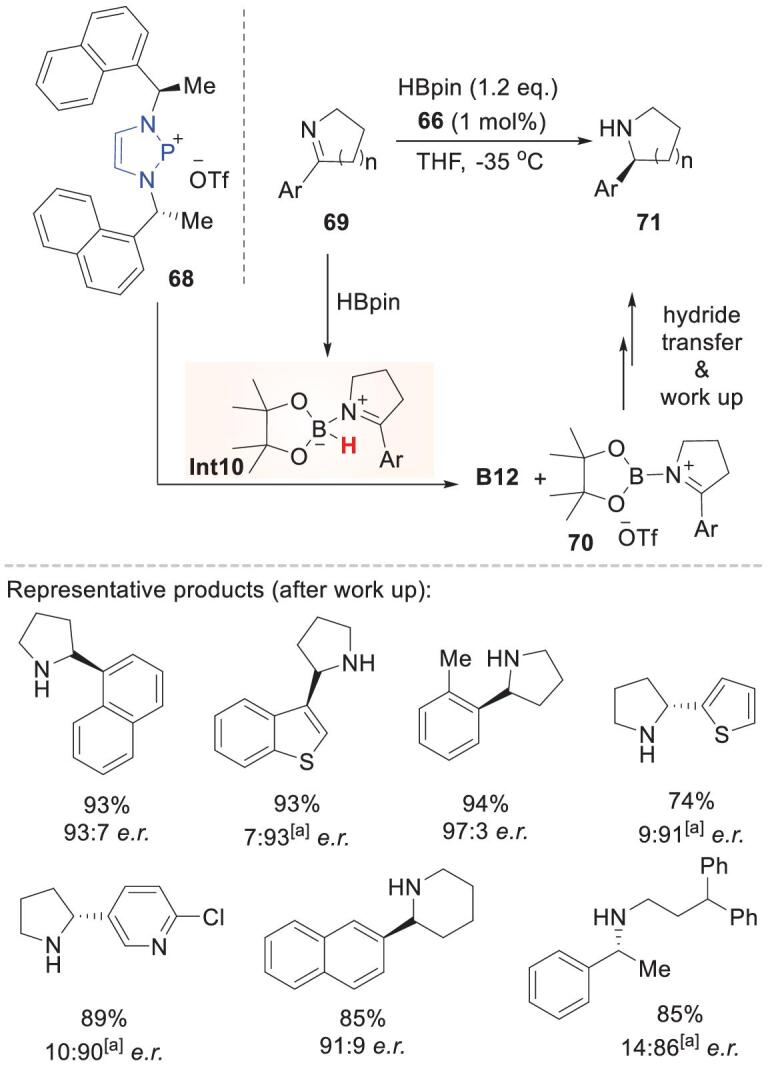
Enantioselective imine reduction catalyzed by chiral phosphenium ion **68**. Enantiomer of **68** was used as the catalyst.

In the above examples, **62** and **65** are both neutral alkoxydiazaphospholenes. Recently, Speed and coworkers used the phosphenium cation **68** to achieve asymmetric reduction. Cyclic imines can be reduced by HBpin with good er values (around 90 : 10) at a catalyst loading of 1 mol% (Scheme 18) [67]. This reaction has good compatibility with imines bearing reducible aromatic heterocycles. A plausible mechanism was proposed for phosphenium-catalyzed pyridine reductions, in which **68** abstracts a hydride from the pinacolborane–imine adduct **Int10** and then redelivers the hydride to the resultant imineborenium **70**. HBpin acts as a Lewis acid in imine activation; therefore, relatively basic imines are preferred. This reactivity complements borane-based frustrated Lewis pair catalysis, in which electron-deficient substrates are needed to avoid product inhibition of electrophilic borane catalysts.

## RADICAL REACTIONS OF NHPs

**Scheme 19.**
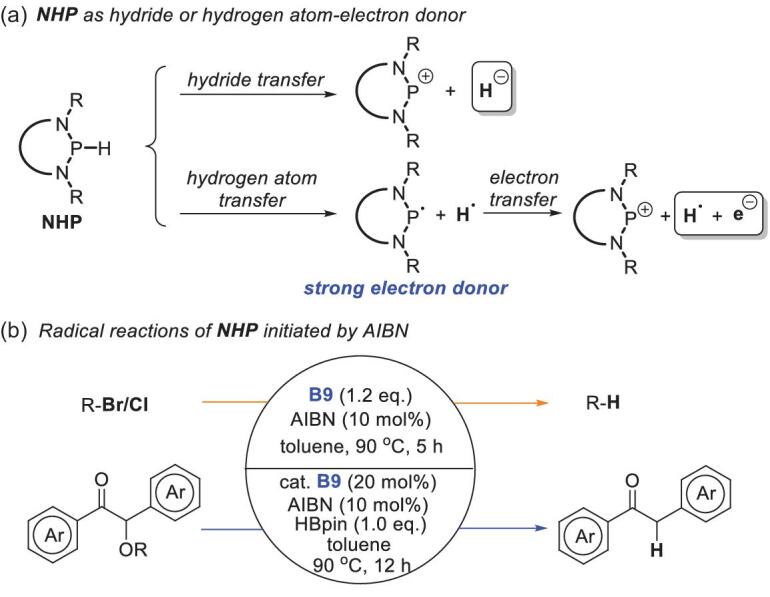
(a) Two pathways for hydride transfer of NHPs and (b) radical reactions of NHPs initiated by azodiisobutyronitrile.

The reactions discussed above mainly depend on the hydridic reduction ability of NHPs. On the basis of our recent findings that NHPs can serve as good hydrogen-atom donors and their corresponding phosphinyl radicals are excellent electron donors [27], we envisioned that the hydride ion of NHPs could be transferred in a multistep mechanism (Scheme 19a), i.e. the hydrogen-atom and electron transfer. This alternative pathway might provide a route to previously inaccessible reactivity in the hydridic reduction of substrates. Our group verified this assumption by using azodiisobutyronitrile as a radical initiator to trigger the initial hydrogen-atom transfer. As expected, the produced phosphinyl radials showed high electron donor activity and enabled hydrodebromination/dechlorination reactions of aryl and alkyl halides [68], and chemoselective cleavage of the α-C–O bonds in α-carboxy ketones [69]. Similar ra-dical reactivity was reported by Speed and coworkers in their bis(diazaphospholene) system [70].

## CONCLUSION AND OUTLOOK

In summary, we have outlined recent progress in NHP chemistry, with special attention to reactivity studies and synthetic applications. The recently disclosed NHP hydricity, as a surrogate for metal-based hydricity, has opened up a new avenue to main-group hydrides, and complements conventional P–H protonic reactivity. Exploitation of new NHPs with greater structural diversity and expansion of their application scope to more challenging substrates will be mainstream in the future. A systematic investigation of the structure–reactivity relationships of NHPs is needed to lay the foundation for this chemistry. Our recent thermodynamic and kinetic studies have shown the ability of NHPs to act as hydrogen-atom donors and of their corresponding phosphinyl radicals to act as potent electron donors. Based on this, we investigated some preliminary applications in electron-transfer-initiated radical reductions. This is a potentially promising area for exploring NHP radical chemistry, particularly in combination with contemporary photocatalytic and electrocatalytic techniques.
